# Highlights of the 12th International Workshop on Adverse Drug Reactions and Co-Morbidities in HIV, London, November 2010

**DOI:** 10.1186/1758-2652-13-S4-O38

**Published:** 2010-11-08

**Authors:** M Schambelan

**Affiliations:** 1 San Francisco General Hospital, Div. of Endocrinology, San Francisco, USA

## 

This presentation will review highlights from the 12th International Workshop on Adverse Drug Reactions and Co-morbidities in HIV that will be held in London on 4-6 November 2010. As in the past, the meeting will consist of plenary lectures by experts from outside the field of HIV medicine as well as oral presentations and posters selected from abstracts submitted by attendees. The plenary speakers for this year's meeting include John Adams (vit D insufficiency), Remy Burcellin (microbial translocation), Kenneth Feingold (TLRs and lipid metabolism), Rolf Jäger (neuroimaging), Cliff Rosen (bone and fat) and Heiner Wedemeyer (ADRs in HBV treatment.). Abstract categories include: adipocyte biology, insulin resistance, aging and associated disorders, lipid metabolism, body composition, liver disease and hepatotoxicity, bone metabolism and toxicities, mitochondrial disorders, cancer in patients with HIV/AIDS, neurocognitive disorders, cardiovascular disease, renal toxicities, clinical management of ADRs, other toxicities and hepatitis.

